# Ruxolitinib in GvHD (RIG) study: a multicenter, randomized phase 2 trial to determine the response rate of Ruxolitinib and best available treatment (BAT) versus BAT in steroid-refractory acute graft-versus-host disease (aGvHD) (NCT02396628)

**DOI:** 10.1186/s12885-018-5045-7

**Published:** 2018-11-19

**Authors:** Nikolas von Bubnoff, Gabriele Ihorst, Olga Grishina, Nadine Röthling, Hartmut Bertz, Justus Duyster, Jürgen Finke, Robert Zeiser

**Affiliations:** 1 Department of Hematology, Oncology and Stem Cell Transplantation, Medical Center, Faculty of Medicine, University of Freiburg, Hugstetter Str. 55, D-79106 Freiburg, Germany; 2 Clinical Trials Unit, Medical Center, Faculty of Medicine, University of Freiburg, Freiburg, Germany; 3 German Cancer Consortium (DKTK), partner site Freiburg, Freiburg, Germany; 4 German Cancer Research Center (DKFZ), Heidelberg, Germany; 5 University Medical Center Göttingen, Göttingen, Germany

**Keywords:** Steroid-refractory GvHD, Ruxolitinib, JAK inhibitor, Biomarkers, Inflammatory cytokines

## Abstract

**Background:**

Graft-versus-Host Disease (GvHD) causes significant morbidity and mortality in patients after allogeneic stem cell transplantation. Donor T-cells cause inflammation and tissue damage in GvHD target organs such as liver, gut and skin. Cytokine receptor associated kinases JAK1 and JAK2 are critical for inflammatory cytokine response in GvHD. Ruxolitinib is a small molecule inhibitor of JAK1 and JAK2. Preliminary data indicated substantial clinical activity in patients with steroid-refractory (SR) acute and chronic GvHD.

**Methods:**

The RIG-study is an investigator-initiated open-label, multicenter, prospective randomized controlled two-arm phase 2 study, comparing the efficacy of ruxolitinib and best available treatment (BAT) versus BAT in steroid-refractory acute GvHD (SR-aGvHD). Patients with acute skin, intestinal or liver GvHD > grade 1 and failure of previous treatment are eligible. The trial aims to include 160 patients who will be randomized in a 1:1 ratio and stratified by GvHD grade (≤ grade 3 versus grade 4) and number of previous immunosuppressive treatments (≤ 3 versus ≥4). The primary endpoint is the overall response rate at day 28, defined as: Improvement of at least one stage in the severity of acute GvHD in one organ without deterioration in any other organ, or disappearance of any GvHD signs from all organs without requirement for new systemic immunosuppressive treatment. Secondary objectives include time to response, overall survival, event-free survival, non-relapse mortality (NRM), failure-free survival, graft failure rates, quality of life and changes in serum levels of pro-inflammatory cytokines and GvHD-related biomarkers.

**Discussion:**

This randomized prospective trial will provide further evidence if the retrospectively collected data demonstrating activity of ruxolitinib for SR-aGvHD can be reproduced. A major advantage of ruxolitinib might be the limited and predictable toxicity profile compared to other immunosuppressive therapies that mainly includes viral reactivation and cytopenias. This trial will establish candidate biomarkers to predict and monitor responses to ruxolitinib. As a next step ruxolitinib might be tested upfront against steroids or in a preemptive manner to prevent GvHD to occur.

**Trial registration:**

NCT02396628 (registration date 17.07.2015); DRKS00007939 (registration date 26.03.2015).

**Electronic supplementary material:**

The online version of this article (10.1186/s12885-018-5045-7) contains supplementary material, which is available to authorized users.

## Background

Steroid-refractory GvHD constitutes the major factor for morbidity and accounts for up to one third of deaths in patients after allogeneic stem cell transplantation [1]. For these patients, there is currently no established standard treatment. Available treatments such as mycophenolate mofetil (MMF), etanercept, daclizumab, basiliximab or Anti-T lymphocyte globulin (ATG) display limited clinical activity with response rates of 20–40% [2]. The anti-CD52 antibody alemtuzumab has been reported to induce high response rates (70%) in patients with aGvHD but however, high rates of infectious complications such as CMV reactivation, bacterial infection, and invasive aspergillosis preclude the broad application of alemtuzumab in steroid refractory GvHD [3–5]. Thus there is a high medical need for novel, active treatments in patients with SR-aGVHD.

The pathophysiological hallmark of GvHD after allogeneic hematopoietic stem cell transplantation (allo-HCT) is an allogeneic donor T cell response against recipient antigens. This process is aggravated by increased processing and presentation of host antigens by donor antigen presenting cells (APCs) following conditioning treatment [6–8]. The allogeneic T cell response leads to inflammation, tissue damage and fibrosis and is mediated by extensive production of inflammatory cytokines such as IL-1, IL-2R, IL-6 and TNF [2]. The signal transmission of inflammatory cytokines in effector cells requires activation of specialized kinases from the family of the Janus kinases. These kinases, JAK1, 2 and 3 are linked to cytokine receptors, and are activated upon binding of the cytokine to the receptor of the inflammatory effector [6, 7]. The development of GvHD in humans is preceded by a dramatic change in the intestinal microflora [2, 9], which could be in part due to a massive influx of neutrophils into the intestinal immune compartment [10] because neutrophils can release proteases and antimicrobial peptides [11] that affect bacteria. This neutrophil infiltration was also observed at late time points after allo-HCT in patients with active GvHD [12]. It was shown that neutrophil activation is dependent on JAK 1/2 signaling [13, 14].

The JAK1/2 kinase inhibitor ruxolitinib (INC424) was approved for intermediate or high-risk myelofibrosis in 2011 and for polycythemia vera with an inadequate response to or intolerance to hydroxyurea in 2014. In myelofibrosis, ruxolitinib lead to sustained clinical remissions with regard to constitutional symptoms, weight loss and spleen size in the majority of treated patients [15]. Of note, clinical responses correlated with a marked reduction in inflammatory plasma cytokines [16]. Importantly, cytokines down-regulated by ruxolitinib in patients with myelofibrosis correspond to inflammatory effectors that mediate tissue damage and inflammation in GvHD. These are mainly the cytokines IL-1, IL-6, TNF and IFN-γ [8, 16].

### Rationale for the trial

Since ruxolitinib suppresses the JAK1/2 dependent cytokine response, we hypothesized that ruxolitinib might attenuate the cytokine mediated inflammatory tissue damage in GvHD and thus might favorably affect the severity and course of GvHD after allo-HCT. In vitro, in an allogeneic system (major mismatch mixed-lymphocyte reactions) we demonstrated that co-incubation with ruxolitinib strongly suppressed both the proliferation of allogeneic T cells and the production of inflammatory cytokines [17]. Using a very aggressive major mismatch mouse model of aGvHD (C57BL/6 on BALBc) ruxolitinib treatment significantly prolonged survival of animals [17]. In addition, in these animals a reduced weight loss was shown, significantly reduced histopathological GvHD severity, suppression of inflammatory cytokines in the serum and a reduction of donor T cells in GvHD target organs such as the intestines. Moreover, we observed that ruxolitinib led to increased frequencies of FoxP3+ regulatory T cells. This cell type was previously shown to lead to long-lasting tolerance [18] as compared to the short-term immunosuppression achieved by conventional medication for GvHD.

Because of this strong evidence of activity of ruxolitinib in animal models, we treated six patients with acute and chronic GvHD with ruxolitinib in a pilot trial [17]. All patients responded with respect to clinical GvHD symptoms and serum levels of pro-inflammatory cytokines. To extend these preliminary findings, we have collected data from a total of 19 stem cell transplant centers in Europe and United States. In this analysis we reported outcome data from 95 patients who received ruxolitinib for corticosteroid-refractory GvHD (skin, mucosa, intestine, liver, lung, musculoskeletal) between 01/2012 and 04/2015 [19]. Patients were classified as having acute (*n* = 54) or chronic (*n* = 41) GvHD. The median number of previous GvHD-therapies was 3 for aGvHD (range: 1–7) and 3 for chronic GvHD (range: 1–10). The overall response rate was 81.4% (44/54) in aGvHD comprising 25 CRs (46%). In cGvHD the overall response rate was 85% (35/41). Clinical improvement was at a median time to response of 1.5 (1–10) weeks and 3 (1–25) weeks after initiation of ruxolitinib treatment in acute and chronic GvHD, respectively. GvHD relapsed in 6.8% (3/44) and 5.7% (2/35) of the ruxolitinib-responsive patients with acute or chronic GvHD. In this analysis, the median follow-up was 22 (3–98) and 25 (2–112) weeks for acute or chronic GvHD patients, respectively. Cytopenias (anemia, leukopenia or thrombocytopenia) and Cytomegalovirus (CMV) reactivation were the most frequently observed adverse events during the time of ruxolitinib treatment occurring in both acute (30/54, 55.5% and 18/54, 33.3%) or chronic (7/41, 17% and 6/41, 14.6%) GvHD patients [19]. Cytopenias had preceded ruxolitinib treatment in 51.8% (28/54) and 14.6% (6/41) of the patients with acute or chronic GvHD. Relapse of the underlying malignancy occurred in 9.2% (5/54) and 2.4% (1/41) of the patients with acute or chronic GvHD, respectively. With a median follow up of 19 months (aGvHD) and 24 months (cGvHD), the 1-year overall survival (OS) was 62.4% for SR-aGvHD (CI: 49.4–75.4%) and 92.7% SR-cGvHD (CI: 84.7–100%) [20]. The estimated median OS was 18 months for aGvHD and not reached for cGvHD patients. The median duration of ruxolitinib treatment was 5 and 10 months for patients with SR-aGvHD and SR-cGvHD, reflecting the different biology of the diseases. 22/54 (41%) SR-aGvHD patients and 10/41 (24%) SR-cGvHD patients had an ongoing response, were free of any immunosuppression and free of ruxolitinib. In responders, GvHD-relapse or GvHD-progression was observed in 14/45 (31%) and 13/36 (36%) patients with SR-aGvHD and SR-cGvHD. Responses to re-treatment with ruxolitinib or any immunosupressive therapy was seen in 11/14 (78%) and 11/13 (86%) patients with SR-aGvHD and SR-cGvHD, respectively. These findings indicated that patients with SR-aGvHD and SR-cGvHD may benefit long-term from ruxolitinib treatment with an OS that is relatively high for steroid-refractory GvHD.

In addition to these data, a prospective clinical trial testing the effects of the JAK1 specific inhibitor itacitinib (INCB039110) in patients with steroid refractory aGvHD indicated an acceptable safety profile [21]. This trial tested the effect of 2 different doses (200 mg and 300 mg PO daily). The reported preliminary ORRs were 83% for first-line therapy patients and 64% for steroid refractory patients.

### Design

Our retrospective analysis demonstrated that ruxolitinib was active and could be safely administered in patients with steroid-refractory GvHD. Based on these data, this study will prospectively evaluate whether ruxolitinib as add-on to BAT is superior to BAT alone in SR-aGvHD. BAT follows current recommendations of the Onkopedia/DGHO guidelines for treatment of aGvHD [22].

**Fig. 1:**
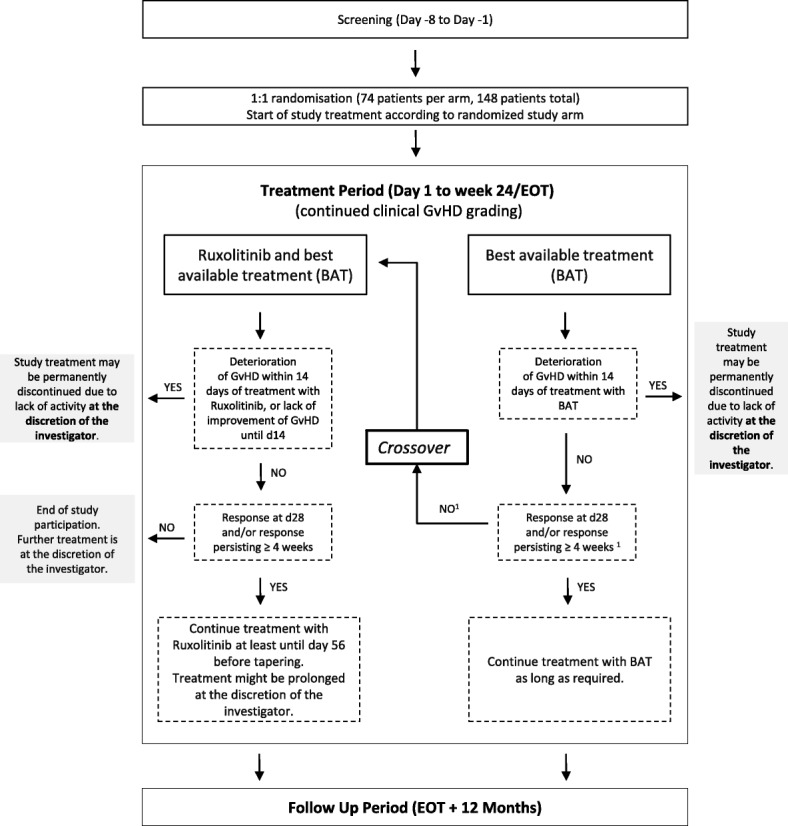
Trial diagram of the RIG-study

The RIG-study is an open-label, multicenter, randomized two-arm phase II study in which patients are randomized to ruxolitinib and BAT versus BAT (Fig. 1). It is planned to randomize 160 patients for the study. Internet-based randomization to one of the two treatment groups is performed in a 1:1 ratio stratified by aGvHD grade (≤ grade 3 versus grade 4) and number of previous immunosuppressive treatments (≤ 3 versus ≥4). Patients will be recruited in 11 transplantation centers in Germany located in Berlin, Bonn, Cologne, Dresden, Freiburg, Hamburg-Eppendorf, Heidelberg, Homburg, Marburg, Munich, and Würzburg. All participating centers are highly experienced in the treatment of patients with aGvHD and stem cell transplantation.

### Study objectives and endpoints

#### Primary endpoint

The primary objective of this trial is to evaluate the efficacy of ruxolitinib added to BAT as compared to BAT alone at day 28 after randomization. The primary endpoint is the overall response rate (ORR) at day 28 defined as proportion of patients in each arm demonstrating partial (PR) or complete response (CR) without requirement for additional systemic immunosuppressive therapy (IST) at day 28 after randomization. PR and CR are defined as improvement of at least one stage in the severity of aGvHD in one organ without deterioration in any other organ (PR) or disappearance of any GvHD signs from all organs (CR) without requirement for addition of new IST, as compared to time of randomization.

#### Secondary endpoints

- ORR at day 14
- Proportion of patients in each arm who achieve a PR or CR without requirement for additional systemic IST, persisting for at least 4 consecutive weeks within 8 weeks after randomization.
- Time to response, defined as time from randomization to the date of first documentation PR or CR. Death or relapse/progression of underlying hematologic disease without prior response will be considered to be a competing event. Comparison of treatment arms is conducted until day 28 only, as crossover to ruxolitinib is offered to non-responders then.
- Rate of patients with treatment failure until day 28 after randomization.
- Duration of response, assessed for responders only by calculating the time from first response to the date of first observation of aGvHD relapse/progression or the date of additional IST for GvHD. Onset of chronic GvHD (cGvHD) or death without prior observation of aGvHD relapse or progression is considered to be a competing events, and observations where neither occurred are treated as censored observations.
- Overall survival (OS) defined as time from randomization to the date of death from any cause.
- Event-free survival (EFS) defined as the time from randomization to the date of recurrence of underlying hematologic disease, graft failure or death due to any cause
- NRM defined as the time from randomization to the date of death not preceded by hematologic disease recurrence. Hematologic disease recurrence / progression are considered to be a competing event.
- Failure-free survival (FFS) is defined as the time from randomization to recurrence of underlying hematologic disease, graft failure, NRM or addition of new systemic IST for GvHD. Onset of cGvHD is considered as a competing event.
- Rate of patients in whom failure to taper steroids below methylprednisolone dose of 0.5 mg/kg/day (or equivalent < 0.6 mg/kg/day of prednisone /prednisolone) was observed within 56 days after randomization OR addition of any new IST for GvHD due to failure to taper steroids below methylprednisolone dose of 0.5 mg/kg/day (or equivalent < 0.6 mg/kg/day of prednisone/prednisolone)
- Rate of patients discontinued ruxolitinib treatment for at least 4 weeks.
- Discontinuation rate of steroid therapy for at least 4 weeks.
- ORR at day 14 in this group, defined as proportion of patients demonstrating PR or CR without requirement for additional systemic IST at day 14 after date of cross over from BAT alone to ruxolitinib/BAT.
- ORR at day 28 in this group, defined as proportion of patients demonstrating PR or CR without requirement for additional systemic IST at day 28 after date of cross over from BAT alone to ruxolitinib/BAT.
- Proportion of patients who achieve a PR or CR without requirement for additional systemic IST, persisting for at least 4 consecutive weeks within 8 weeks after the date of cross over from BAT only to ruxolitinib/BAT.
- Cumulative steroid dose until day 56.
- Reduction of GvHD blood biomarkers on day 8 randomization.
- Relapse rate of underlying hematologic disease, calculated as incidence rate, time from randomization to the date of relapse diagnosis. Death without prior relapse will be considered as a competing event.
- Graft failure incidence rates, calculated as time from randomization to the date of graft failure diagnosis. Death without prior graft failure will be considered as a competing event.
- Quality of life (QoL): EORTC QLQ-C30 and EORTC QLQ-C29.
- Time from randomization to the start date of any cGvHD. Hematologic relapse/progression and death without prior cGvHD will be considered as competing events.
- Type, frequency, severity and relationship of adverse events to investigational product, engraftment, infections.
- Frequency of CMV reactivation.
- Number of nights in hospital.

#### Target population

Adult male and female patients with acute skin, intestinal or liver GvHD according to published criteria of the International Bone Marrow Transplant Registry and lack of response to corticosteroid treatment will be enrolled. In GvHD, a gender distribution of 2:1 (male to female) is expected. Gender or age do not impact on response and are not considered risk factors. Therefore we will not stratify for gender or age in this study. Written informed consent must be obtained from the patient before starting any study related procedures. After all screening evaluations have been performed patients meeting all inclusion and none of the exclusion criteria can be included into the study.

#### Inclusion criteria

For inclusion into the study, patients must fulfill all of the following criteria:

1. Acute skin, intestinal or liver GvHD > grade 1 according to standard criteria
2. Histological confirmation in case of acute intestinal GvHD
3. Age ≥ 18 years
4. Failure of previous treatment, defined as presence of at least one of the following criteria:
  Treatment with prednisone/prednisolone/methylprednisolone in a dose of at least 2 mg/kg and lack of response after at least 7 days treatment
  Treatment with prednisone/prednisolone /methylprednisolone in a dose of at least 2 mg/kg and progression after at least 3 days of treatment
  Failure to taper the prednisone/prednisolone dose to 0.6 mg/kg/day or methylprednisolone dose to < 0.5 mg/kg/day
5. Written informed consent
6. Ability to understand the nature of the study and the study related procedures and to comply with them

#### Exclusion criteria

Patients are not eligible for this study if they fulfill one or more of the following exclusion criteria:

1. Uncontrolled underlying disease
2. Active bleeding
3. Absence of clinical signs of acute GvHD [23]
4. Diagnostic or distinctive clinical signs of chronic GvHD [1]
5. Uncontrolled bacterial, viral or fungal infection
6. Any previous JAK2 inhibitor treatment prior to study enrolment, except ruxolitinib given prior to the allogeneic stem cell transplantation
7. Known Hypersensitivity to ruxolitinib or any of the excipients
8. Known positivity for HIV, Hepatitis B or Hepatitis C at the time of screening.
9. Female patients who are pregnant or breast feeding
10. Concomitant use of any other investigational drug within the last thirty days before the start of this study

### Study treatment

#### Ruxolitinib treatment

Ruxolitinib treatment will be administered for 6 months or as long as the patient experiences benefit from treatment with ruxolitinib. In this case treatment might be prolonged at the discretion of the investigator. In patients with CR or stable PR (≥ 4 weeks) and off steroids, ruxolitinib will be tapered. Patients randomized to ruxolitinib and BAT who do not meet the primary endpoint at day 28 will discontinue ruxolitinib and will be treated at the discretion of the investigator. In case of relapse of underlying malignancy, the study treatment will be permanently discontinued.

#### Best available treatment (BAT)

Prior to randomization, the most appropriate systemic BAT treatment will be selected as investigators choice in a patient-individualized manner based on the Onkopedia/DGHO guidelines for treatment of acute GvHD as of January 2017 [22].

### Data collection and follow-up

#### Screening assessment

The following examinations and laboratory tests will be performed at screening within 8 days and day − 1 prior to first intake of medication; physical examination, current disease status, clinical GvHD grading, standard laboratory tests, CMV PCR, QoL, evaluation of concomitant and GvHD medication. When screening assessments are completed, check and validation of inclusion and exclusion criteria for the study is performed. Detailed information on all screening evaluations is given in Additional file 1: Table S1 and Table S2.

#### Assessments during the treatment phase

Treatment visits are performed weekly from day 1 to week 6; bi-weekly from week 8 to week 12; monthly from week 16 to week 24. If the patient benefit from treatment with ruxolitinib the treatment might be prolonged at the discretion of the investigator. Therefore bi-monthly visits will be scheduled. Treatment visits contain physical examination, clinical GvHD grading, measurements of standard laboratory tests, CMV PCR, QoL, evaluation of concomitant and GvHD medication, assessment of adverse events and evaluation of number of in-hospital days. Detailed information on all assessments during the treatment phase is given in Additional file 1: Table S1 and Table S2.

#### Assessments during the follow-up phase

After end of treatment patients continue with follow up within the study. The following information will be assessed each time the patient visits the site for Follow up visit (every 2 months for 12 months): physical examination, clinical GvHD grading, standard laboratory tests, CMV PCR, QoL, evaluation of concomitant and GvHD medication, assessment of adverse events and evaluation of number of in-hospital days.

#### Statistical planning

The sample size calculation is based on the primary endpoint response (ORR). An expected proportion of 30% for the patients with best available treatment and an expected alternative proportion of 50% for the patients with acute GvHD treated with ruxolitinib is assumed. As there is currently no satisfactory active treatment available for patients with steroid-refractory GvHD, the alternative assumption is based on published data of 60 patients with acute and chronic GvHD treated with ruxolitinib [17, 19] and on recent papers for BAT [3–5]. The study is planned to detect a response difference between treatment arms at a one-sided significance level α = 5% with a power of 1-β = 80%, when the response probabilities are 50% (ruxolitinib) and 30% (BAT). Based on the χ2 Test, 148 patients (74 per group) need to be included in the analysis. A small number of drop-outs not evaluable for the intention-to-treat analysis of the primary endpoint may occur. Further missing response will be evaluated as non-response according to the intention-to-treat principle. This might blur the assumed difference between treatment groups. Therefore we increased the calculated sample size by 8%, and 160 patients will be randomized.

The primary analysis will be based on the full analysis set (FAS) and will be conducted with a logistic regression model of the primary endpoint response to GvHD treatment as defined above. The model will include treatment assignment (ruxolitinib and BAT vs BAT only) and the stratification variables aGvHD grade (≤ grade 3 versus grade 4) and number of previous immunosuppressive treatments (≤ 3 versus ≥4) as covariates, and the primary hypothesis will be tested at a one sided level α = 5% within this model. Odds ratios will be calculated with two-sided 90% confidence intervals (CI), and the null hypothesis will be rejected in favour of the alternative, if the lower bound of the CI for the odds ratio of ruxolitinib and BAT compared to BAT only is above 1. Patients with missing response evaluation will be counted as non-responders. This analysis will be repeated in the per protocol (PP) population as a sensitivity analysis. Additionally, two-sided 95% CI will be reported in order to provide comparability with data from literature.

Binary secondary endpoints (e.g. proportion of patients with CR, proportion of patients dis-continuing treatment) will be estimated as rates and presented with exact two-sided 95% confidence intervals derived from the binomial distribution. Furthermore, the same type of logistic model using treatment assignment, aGvHD grade and number of immunosuppressive treatments as covariates will be applied.

OS and EFS probabilities will be estimated and displayed using the Kaplan Meier method.

The analysis of secondary time-to-event endpoints with competing risks (e.g. time to response, response duration, time to cGvHD development) will be performed by means of the Aalen Johanson estimator for the calculation of cumulative incidence rates. Appropriate Regression models (Cox models for OS, EFS, Fine and Gray models for endpoints with competing events) will be applied. For FFS, different types of failure will be analyzed separately, considering the other types of failure and onset of cGvHD as competing events. For evaluating QOL, scores will be calculated according to the EORTC QLQ-C30 Scoring Manual [24]. The scores at screening will be summarized and for different time points during follow-up, differences of the scores to the screening scores will be calculated in FAS and the PP set.

#### Data safety monitoring board (DSMB)

A Data Safety Monitoring Board (DSMB) is established to supervise the conduct of the trial and will be responsible for recommendations for early termination, modifications or continuation of the trial, if necessary.

## Discussion

Retrospective data suggest that JAK1/2 inhibition with ruxolitinib can induce clinically meaningful and ongoing responses in heavily pretreated patients with acute and chronic steroid-refractory GvHD. To confirm these observations, single arm (NCT02953678, NCT02997280, NCT02806375) and randomized prospective trials (NCT02913261, NCT03112603) are ongoing. To establish the benefit of ruxolitinib versus best available treatment (BAT), a randomized design is needed. This trial (NCT02396628) is intended to examine ruxolitinib as add-on to BAT versus BAT alone. Thus, previously started GvHD treatments can be continued in addition to ruxolitinib at the discretion of the investigator. There is no limitation of previous and concomitant lines of aGvHD treatments, since patients in our retrospective series had a median number of three (range: 1–7) previous GvHD therapies [19]. This trial allows cross-over from BAT to BAT and ruxolitinib for patients receiving BAT not achieving the primary endpoint at day 28, with loss of response after day 28 or failure to taper steroids. However in both arms, in case of deterioration of GvHD within the first 14 days or lack of improvement, treatment may be permanently discontinued at the discretion of the investigator. In this case, the protocol recommends individual case-by-case discussion with the sponsor. Patients in the ruxolitinib arm not meeting the primary endpoint at day 28 will discontinue ruxolitinib and will be treated at the discretion of the investigator, although in our retrospective analysis we observed responses in aGvHD up to week eleven of treatment. The duration of treatment will be 6 months or as long as the patient experiences benefit from ruxolitinib treatment. In case of CR or stable PR (≥ 4 weeks) and off steroids, ruxolitinib is slowly tapered starting at day 56. In case of reappearance of GvHD signs during tapering or after tapering has been completed, the dose may be increased or treatment with ruxolitinib may be reinstituted. Hematological adverse events will be recorded but however, dose interruptions and dose reductions during treatment due to hematological adverse events are optional and as required in the investigators opinion. Also, baseline cytopenias are not prohibitive for inclusion. The protocol includes dose modifications for drug-related, non-hematological adverse events that are not GvHD related. As a unique feature of this trial, study drug is provided by regular prescription beyond approved indication. Accounting of study drug was approved by the Gemeinsame Bundesausschuss (GBA) according to § 35c Abs. 2 SGB V.

## Conclusion

The RIG-study is an open-label, multicenter, randomized two-arm phase 2 study for patients with steroid-refractory aGvHD. Patients are randomized to ruxolitinib and BAT versus BAT. This trial will prospectively evaluate whether ruxolitinib as add-on to BAT is superior to BAT alone in steroid-refractory aGvHD.

## Additional file

Additional file 1: **Table S1**. Visit schedule treatment phase prior to cross over. **Table S2**. Visit schedule treatment phase after cross over from BAT only to Ruxoltinib/BAT. (DOCX 144 kb)

## Abbreviations

aGvHD: Acute Graft-versus-Host Disease
APC: Antigen presenting cell
ATG: Anti-T-lymphocyte Globulin
BAT: Best available treatment
cGvHD: Chronic Graft-versus-Host Disease
CMV: Cytomegalovirus
CR: Complete Response
DGHO: Deutsche Gesellschaft für Hämatologie und Onkologie
DLI: Donor lymphocyte infusion
EORTC: European Organization for Research and Treatment of Cancer
FAS: Full analysis set
GCP: Good Clinical Practice
GvHD: Graft-versus-Host Disease
HCT: Hematopoietic stem cell transplantation
HIV: Human Immunodeficiency Virus
IBMTR: International Bone Marrow Transplant Registry
IL-6: Interleukin 6
IST: Immunosuppressive therapy
LDH: Lactate dehydrogenase
MMF: Mycophenolate mofetil
NIH: National Institutes of Health
NRM: Non-relapse mortality
ORR: Overall response rate
OS: Overall survival
PCR: Polymerase Chain Reaction
PR: Partial Response
QLQ: Quality of Life Questionnaire
SAE: Serious Adverse Events
SD: Stable disease
SGB: Sozialgesetzbuch (“Code of Social Law”)
sIL-2R: Soluble interleukin-2 receptor
SR-aGVHD: Steroid refractory acute GvHD
TIM-3: T cell lg and mucin domain 3 (TIM-3) receptor
TNF: Tumor necrosis factor
WHO: World Health Organization
