# Supplementary material for: Ruxolitinib in GvHD (RIG) study: a multicenter, randomized phase 2 trial to determine the response rate of Ruxolitinib and best available treatment (BAT) versus BAT in steroid-refractory acute graft-versus-host disease (aGvHD) (NCT02396628)
Source: BMC Cancer. 2018 Nov 19;18:1132. doi: 10.1186/s12885-018-5045-7 (PMC6245867; doi:10.1186/s12885-018-5045-7)
Supplement: Supplementary file 1 — Table S1. Visit schedule treatment phase prior to cross over. Table S2. Visit schedule treatment phase after cross over from BAT only to Ruxoltinib/BAT. (DOCX 144 kb) [file 12885_2018_5045_MOESM1_ESM.docx]

Supplementary Table 1: Study procedure and Flow Chart (treatment phase prior to cross over)

| **Assessments** | **Screening**  **Examination**  -8 days to day -1 | **Weekly treatment period**  from day 1 to week 6  *(± 2 days)* | | | | | | | | | | **Biweekly**  **treatment**  **period**  from week 8 to  week 12 *(± 2 days)* | | | **Monthly**  **treatment period**  from week 16 to  week 24  *(± 2 days)* | | | **Additional**  **treatment**  **phase^[[1]](#endnote-2)^**  every two  months  *(± 14 days)* | **Follow Up**  every two  months for 12 months  *(± 14 days)* | | | | | **End of**  **Study** |
| --- | --- | --- | --- | --- | --- | --- | --- | --- | --- | --- | --- | --- | --- | --- | --- | --- | --- | --- | --- | --- | --- | --- | --- | --- |
|  |  | Week 1,  day 1 | Week 2,  day 8 | | Week 3,  day 15 | | Week 4,  day 22 | | Week 5,  day 29 | | Week 6,  day 36 | Week 8,  day 50 | Week 10,  day 64 | Week 12,  day 78 | Week 16,  day 106 | Week 20,  day 134 | Week 24,  day 162 |  | Month 2 | Month 4 | Month 6 | Month 8 | Month 10 | Month 12 |
| Informed Consent | x |  |  | |  | |  | |  | |  |  |  |  |  |  |  |  |  |  |  |  |  |  |
| Patient eligibility | x |  |  | |  | |  | |  | |  |  |  |  |  |  |  |  |  |  |  |  |  |  |
| Current disease status^[[2]](#endnote-3)^ | x |  |  | |  | |  | |  | |  |  |  |  |  |  |  |  |  |  |  |  |  |  |
| Randomisation | x |  |  | |  | |  | |  | |  |  |  |  |  |  |  |  |  |  |  |  |  |  |
| **Ruxolitinib/BAT or BAT only** |  | ***continues every day from day 1*** | | | | | | | | | | | | | | | | x |  |  |  |  |  |  |
| Physical examination | x | x | | x | | x | | x | | x | x | x | x | x | x | x | x | x | x | x | x | x | x | x |
| Weight | x | x | | x | | x | | x | | x | x | x | x | x | x | x | x | x | x | x | x | x | x | x |
| Clinical GvHD grading^[[3]](#endnote-4)^ | x | x | | x | | x | | x | | x | x | x | x | x | x | x | x | x | x | x | x | x | x | x |
| Gut biopsy (mandatory)^[[4]](#endnote-5)^ | x |  | |  | |  | |  | |  |  |  |  |  |  |  |  |  |  |  |  |  |  |  |
| Skin biopsy (optional)^[[5]](#endnote-6)^ | x |  | |  | |  | |  | |  |  |  |  |  |  |  |  |  |  |  |  |  |  |  |
| Laboratory(Haematology, clinical chemistry)^[[6]](#endnote-7)^ | x | x | | x | | x | | x | | x | x | x | x | x | x | x | x | x | x | x | x | x | x | x |
| CMV PCR^[[7]](#endnote-8)^ | x | x | | x | | x | | x | | x | x | x | x | x | x | x | x | x | x | x | x | x | x | x |
| Serum biomarker sIL-2R, IL-6, TNF (local)^[[8]](#endnote-9)^ | x | x | | x | |  | | x | |  |  | x |  |  | x |  | x | x | x | x | x | x | x | x |
| Serum sample ST2, TIM3 (central)^[[9]](#endnote-10)^ | x | x | | x | |  | | x | |  |  | x |  |  | x |  | x | x | x | x | x | x | x | x |
| EDTA samples (central) | x |  | | x | |  | |  | |  |  |  |  |  |  |  |  |  |  |  |  |  |  |  |
| EORTC QLQ-C30 and QLQ-HDC29 | x | x | | x | | x | | x | | x | x | x | x | x | x | x | x | x | x | x | x | x | x | x |
| Concomitant Medication^[[10]](#endnote-11)^ | x | x | | x | | x | | x | | x | x | x | x | x | x | x | x | x | x | x | x | x | x | x |
| Adverse Events^[[11]](#endnote-12)^ |  | x | | x | | x | | x | | x | x | x | x | x | x | x | x | x | x | x | x | x | x | x |
| Monitoring of in-hospital days^[[12]](#endnote-13)^ |  | x | | x | | x | | x | | x | x | x | x | x | x | x | x | x | x | x | x | x | x | x |

Supplementary Table 2: Study procedure and Flow Chart (treatment phase after cross over from BAT only to Ruxoltinib/BAT)

| **Assessments** | **Weekly treatment period**  from day 1 to week 6  *(± 2 days)* | | | | | | **Biweekly**  **treatment**  **period**  from week 8 to  week 12 *(± 2 days)* | | | **Monthly**  **treatment period**  from week 16 to  week 24 *(± 2 days)* | | | **Additional**  **treatment**  **phase***^[[13]](#endnote-14)^*  every two  months  *(± 14 days)* | **Follow Up**  every two  months for 12 months  *(± 14 days)* | | | | | **End of**  **Study** |
| --- | --- | --- | --- | --- | --- | --- | --- | --- | --- | --- | --- | --- | --- | --- | --- | --- | --- | --- | --- |
|  | Week 1,  day 1 | Week 2,  day 8 | Week 3,  day 15 | Week 4,  day 22 | Week 5,  day 29 | Week 6,  day 36 | Week 8,  day 50 | Week 10,  day 64 | Week 12,  day 78 | Week 16,  day 106 | Week 20,  day 134 | Week 24,  day 162 |  | Month 2 | Month 4 | Month 6 | Month 8 | Month 10 | Month 12 |
| **Ruxolitinib/BAT** | ***continues every day from day 1*** | | | | | | | | | | | | x |  |  |  |  |  |  |
| Physical Examination and vital signs | x | x | x | x | x | x | x | x | x | x | x | x | x | x | x | x | x | x | x |
| Weight | x | x | x | x | x | x | x | x | x | x | x | x | x | x | x | x | x | x | x |
| Clinical GvHD grading^[[14]](#endnote-15)^ | x | x | x | x | x | x | x | x | x | x | x | x | x | x | x | x | x | x | x |
| Laboratory(Haematology, clinical chemistry)^[[15]](#endnote-16)^ | x | x | x | x | x | x | x | x | x | x | x | x | x | x | x | x | x | x | x |
| CMV PCR^[[16]](#endnote-17)^ | x | x | x | x | x | x | x | x | x | x | x | x | x | x | x | x | x | x | x |
| Serum biomarker sIL-2R, IL-6, TNF (local)^[[17]](#endnote-18)^ | x | x |  | x |  |  | x |  |  | x |  | x | x | x | x | x | x | x | x |
| Serum sample ST2, TIM-3 (central)^[[18]](#endnote-19)^ | x | x |  | x |  |  | x |  |  | x |  | x | x | x | x | x | x | x | x |
| EDTA samples (central) |  | x |  |  |  |  |  |  |  |  |  |  |  |  |  |  |  |  |  |
| EORTC QLQ-C30 and QLQ-HDC29 | x | x | x | x | x | x | x | x | x | x | x | x | x | x | x | x | x | x | x |
| Concomitant Medication^[[19]](#endnote-20)^ | x | x | x | x | x | x | x | x | x | x | x | x | x | x | x | x | x | x | x |
| Adverse Events^[[20]](#endnote-21)^ | x | x | x | x | x | x | x | x | x | x | x | x | x | x | x | x | x | x | x |
| Monitoring of in-hospital days^[[21]](#endnote-22)^ | x | x | x | x | x | x | x | x | x | x | x | x | x | x | x | x | x | x | x |

##

1. Study treatment will be administered for 6 months or as long as the patient experiences benefit from treatment with Ruxolitinib. In this case treatment might be prolonged at the discretion of the investigator. In case of reappearance of GvHD signs after Ruxolitinib has been discontinued, treatment with Ruxolitinib may be reinstituted. At end of treatment the same assessments will be done as month 12. [↑](#endnote-ref-2)
2. Current disease status: CMV Status donor/recipient, previous treatment of the underlying disease including allogeneic transplantation, previous GvHD treatment including GvHD prophylaxis, corticosteroid treatment with duration, dose and response, and earlier infectious complications during induction/consolidation. [↑](#endnote-ref-3)
3. According to Appendix 1 of the protocol. [↑](#endnote-ref-4)
4. Gut biopsy is mandatory but however, can be performed at any time before screening. Thus, time frame for biopsy is NOT limited to day -8 to day -1. [↑](#endnote-ref-5)
5. Skin biopsy is recommended to be performed at any time before screening. Time frame for biopsy is NOT limited to day -8 to day -1. [↑](#endnote-ref-6)
6. Local lab: hematology and clinical chemistry will be performed according to clinical routine. In the CRF only the following parameters will be documented: hemoglobin, leukocyte, platelets and neutrophil count. Laboratory data have to be checked by the investigator and in case of a clinically relevant abnormality an AE has to be recorded on the AE-page in the CRF. [↑](#endnote-ref-7)
7. CMV PCR to be performed once weekly from week 1 to 6, and once every other week from week 8 to 12 and monthly from week 16 to 24. In patients who never reactivated CMV then once every 2 months. In patients with CMV reactivation, it is recommended to determine the CMV copy number twice per week. For CMV monitoring and recommended preemptive treatment see chapter 7.11 of the protocol. [↑](#endnote-ref-8)
8. The following serum biomarkers will be measured locally at the center: sIL2-R, IL-6, TNF. [↑](#endnote-ref-9)
9. Serum sample for ST2 and TIM-3 will be shipped to University Medical Center Freiburg for central assessment (see laboratory manual). [↑](#endnote-ref-10)
10. As Ruxolitinib is a substrate of CYP 3A4, CYP 2C9 and P-Glycoprotein, please check possible drug interactions before prescribing/administration of any new medication. **Administration of fluconazole at daily doses higher than 200 mg is prohibited.** All concomitant tumor-specific or other disease-modifying therapy including “targeted” therapies (other than investigational products) or systemic GvHD treatment administered at any time during the period starting with the signature of the Informed Consent Form (ICF) and ending with the study end, has to be thoroughly documented in the CRFs. For details see section 7.10 of the protocol. [↑](#endnote-ref-11)
11. The AE reporting and documentation period begins with randomization and ends at the end of month 6 (for both treatment arms) or 30 days after last intake of ruxolitinib (whichever occurs later). For patients in the BAT arm who crossed over to Ruxolitinib/BAT the reporting and documentation period ends 30 days after last intake of ruxolitinib. For details regarding SAE reporting and documentation please refer to section 9 of the protocol. [↑](#endnote-ref-12)
12. Inpatient or outpatient status and duration of current and last inpatient hospitalization will be documented in the CRF. [↑](#endnote-ref-13)
13. Study treatment will be administered for 6 months or as long as the patient experiences benefit from treatment with Ruxolitinib. In this case treatment might be prolonged at the discretion of the investigator. In case of reappearance of GvHD signs after Ruxolitinib has been discontinued, treatment with Ruxolitinib may be reinstituted. At end of treatment the same assessments will be done as month 12. [↑](#endnote-ref-14)
14. According to [22]; see Appendix 1 of the protocol. [↑](#endnote-ref-15)
15. Local lab: hematology and clinical chemistry will be performed according to clinical routine. In the CRF only the following parameter will be documented: hemoglobin, leukocyte, platelets and neutrophil count. Laboratory data have to be checked by the investigator and in case of a clinically relevant abnormality an AE has to be recorded on the AE-page in the CRF. [↑](#endnote-ref-16)
16. CMV PCR to be performed once weekly from week 1 to 6, and once every other week from week 8 to 12 and monthly from week 16 to 24. In patients who never reactivated CMV then once every 2 months. In patients with CMV reactivation, it is recommended to determine the CMV copy number twice per week. For CMV monitoring and recommended preemptive treatment see chapter 7.11 of the protocol. [↑](#endnote-ref-17)
17. The following serum biomarkers will be measured locally at the center: sIL2-R, IL-6, TNF. [↑](#endnote-ref-18)
18. Serum sample for ST2 and TIM-3 will be shipped to Medical Center - University of Freiburg for central assessment (see laboratory manual). [↑](#endnote-ref-19)
19. As Ruxolitinib is a substrate of CYP 3A4, CYP 2C9 and P-Glycoprotein, please check possible drug interactions before prescribing/administration of any new medication. **Administration of fluconazole at daily doses higher than 200 mg is prohibited.** All concomitant tumor-specific or other disease-modifying therapy including “targeted” therapies (other than investigational products) or systemic GvHD treatment administered at any time during the period starting with the signature of the Informed Consent Form (ICF) and ending with the study end, has to be thoroughly documented in the CRFs. For details see section 7.10 of the protocol. [↑](#endnote-ref-20)
20. The AE reporting and documentation period begins with randomization and ends at the end of month 6 (for both treatment arms) or 30 days after last intake of ruxolitinib (whichever occurs later). For patients in the BAT arm who crossed over to Ruxolitinib/BAT the reporting and documentation period ends 30 days after last intake of ruxolitinib. For details regarding SAE reporting and documentation please refer to section 9 of the protocol. [↑](#endnote-ref-21)
21. Inpatient or outpatient status and duration of current and last inpatient hospitalization will be documented in the CRF. [↑](#endnote-ref-22)
